# Rab7 Mutants Associated with Charcot-Marie-Tooth Disease Exhibit Enhanced NGF-Stimulated Signaling

**DOI:** 10.1371/journal.pone.0015351

**Published:** 2010-12-09

**Authors:** Soumik BasuRay, Sanchita Mukherjee, Elsa Romero, Michael C. Wilson, Angela Wandinger-Ness

**Affiliations:** 1 Department of Pathology and Cancer Research and Treatment Center, University of New Mexico Health Sciences Center, Albuquerque, New Mexico, United States of America; 2 Department of Neurosciences, University of New Mexico Health Sciences Center, Albuquerque, New Mexico, United States of America; National Institutes of Health, United States of America

## Abstract

Missense mutants in the late endosomal Rab7 GTPase cause the autosomal dominant peripheral neuropathy Charcot-Marie-Tooth disease type 2B (CMT2B). As yet, the pathological mechanisms connecting mutant Rab7 protein expression to altered neuronal function are undefined. Here, we analyze the effects Rab7 CMT2B mutants on nerve growth factor (NGF) dependent intracellular signaling in PC12 cells. The nerve growth factor receptor TrkA interacted similarly with Rab7 wild-type and CMT2B mutant proteins, but the mutant proteins significantly enhanced TrkA phosphorylation in response to brief NGF stimulation. Two downstream signaling pathways (Erk1/2 and Akt) that are directly activated in response to phospho-TrkA were differentially affected. Akt signaling, arising in response to activated TrkA at the plasma membrane was unaffected. However Erk1/2 phosphorylation, triggered on signaling endosomes, was increased. Cytoplasmic phospho-Erk1/2 persisted at elevated levels relative to control samples for up to 24 h following NGF stimulation. Nuclear shuttling of phospho Erk1/2, which is required to induce MAPK phosphatase expression and down regulate signaling, was greatly reduced by the Rab7 CMT2B mutants and explains the previously reported inhibition in PC12 neurite outgrowth. In conclusion, the data demonstrate a mechanistic link between Rab7 CMT2B mutants and altered TrkA and Erk1/2 signaling from endosomes.

## Introduction

Charcot-Marie-Tooth (CMT) disease is a family of inherited disorders that usually results in the degeneration of peripheral sensory and motor neurons [1]–[4]. Disease onset occurs in adolescence or adulthood and is manifested by muscle weakness and loss of fine motor control and sensation in the extremities. The five disease subtypes include autosomal dominant, X-linked and recessive forms that affect the function of Schwann cells and/or neurons and fall into two general groups [2]. Type 1 subtypes affect myelination or cell communication principally in Schwann cells and include CMT1A-B, CMT3 (hypertrophic neuropathy of Dejerine-Sottas), some forms of CMT4A, CMT4B and CMTX [1]. Type 2 subtypes result in axonal degeneration and include CMT2A-G, I-L, and intermediate CMT, DI-CMTB [4]. The similarities in disease manifestation resulting from mutations in over 20 known genes may be ascribed to the extensive interdependence of Schwann cells and neurons, and to the fact that the gene products are operative on common pathways. For example, multiple CMT disease associated genes affect proteins that regulate endocytic membrane transport and thereby impact neurons and Schwann cells in the maintenance of axon viability.

Among the genes associated with CMT disease are those that encode proteins involved in the regulation of endocytosis and growth factor signaling, one of which is the Rab7 GTPase. Rab7 regulates transport of growth factors and other cargo from early to late endosomes and on to lysosomes by controlling membrane budding, cytoskeletal transport and fusion between endocytic compartments. Following nerve growth factor (NGF) stimulation of neurons, Rab7 forms a transient complex with the internalized NGF receptor called TrkA and thereby impacts receptor signaling and neurite outgrowth [5]–[9]. Expression of dominant negative Rab7 prolongs TrkA signaling, increases the activation of downstream Erk1/2 and alters neurite outgrowth [9]. Similarly, ectopic expression of the Rab7 CMT mutants leads to an inhibition of neurite outgrowth in PC12 cells [10]. Given the close association between Rab7 and TrkA in regulating neurite outgrowth, it remains to be clarified how the CMT disease mutants affect the interaction and activation of TrkA. To better understand the underlying mechanisms of CMT it is also of interest to define the impact of these mutations.

There are four mutations in the Rab7 gene that have been identified as disease-causal in CMT2B patients [11]–[13]. All four are missense mutations that change residues L129F, K157N, N161T and V162M lie outside of the nucleotide-binding pocket. Interestingly, however these mutations alter highly conserved amino acids present in all Rab GTPases, suggesting that they are critical to the function of these small GTPases. In contrast, point mutations in or near the nucleotide binding pocket of Rab7 have been well-characterized and have been shown to preferentially stabilize Rab7 in the active (GTP-bound, Rab7Q67L) or inactive (GDP-bound, Rab7T22N) states. In particular, inactivating mutants have significant effect on endocytic trafficking and signaling [5]–[7], [9], [14], [15]. While the Rab7 CMT2B mutants do retain GTP-binding activity they exhibit dysregulated nucleotide binding in vitro [16], [17]. This effect on nucleotide binding is thought to result in increased retention of mutant protein on target membranes leading to the enhanced interaction with a subset of effector proteins [16]. Interestingly however it has been reported that the binding to the effector protein, RILP, that regulates retrograde trafficking of TrkA positive endosomes to the cell body was unaffected by these mutations [17], yet neurite outgrowth was altered [10]. Because the fidelity of endocytic transport from internalization to lysosome delivery and proper coupling to signaling pathways plays a pivotal role in the maintenance of myelination and axonal function of sensory and motor neurons it becomes important to understand the mechanistic details of how the disease specific mutations alter intracellular signaling.

In the present study, we demonstrate that the Rab7 CMT2B mutants increase endosomal TrkA signaling leading to enhanced Erk signaling. The activated Erk proteins predominate in the cytoplasm in lieu of their normal nuclear localization following NGF stimulation of TrkA. These observations provide new insight into the molecular basis for the alterations in neurite outgrowth and axonal viability associated with the CMT disease state.

## Results

### The CMT2B-associated Rab7 mutants interact with TrkA

**Figure 1:**
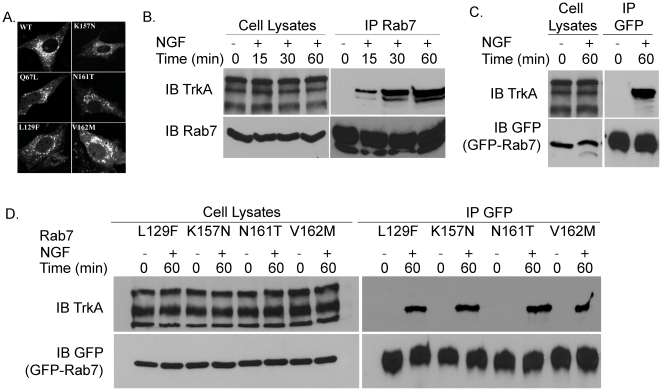
Rab7 CMT2B Mutants are Membrane Bound and Interact with TrkA. (A) BHK cells were transfected with GFP-tagged Rab7 wild-type and CMT2B disease mutants. After 16 h of transfection, cells were fixed and permeabilized by saponin treatment. Enlarged perinuclear vesicular staining was observed for the CMT2B disease mutants. (B) PC12 cells were stimulated with NGF (200 ng/ml) for 15–60 min as indicated. Cells were lysed and samples of cell lysates were immunoblotted for TrkA and Rab7. Lysate samples containing equal amounts of protein were immunoprecipitated (IP) with a pAb directed against Rab7 and immunoblotted for TrkA. Subsequently, the membrane was stripped and reprobed for Rab7. (C) PC12 cells were transfected with wild-type GFP-Rab7 and stimulated with NGF for 60 min. Samples containing equal amounts of protein were immunoprecipitated with antibodies against GFP to isolate the GFP-Rab7 and subsequently immunoblotted for TrkA. The membrane was stripped and reprobed with antibodies against GFP. (D) PC12 cells were transfected with GFP-tagged Rab7 CMT2B disease mutants and stimulated with NGF for 60 min. Equal amounts of lysate samples were immunoprecipitated with antibodies against GFP to precipitate the GFP-Rab7 and subsequently immunoblotted for TrkA. The membrane was stripped and reprobed with antibodies against GFP. (B–D) Representative blots from one of three independent experiments are shown in each case.

The interaction of Rab7 with the receptor tyrosine kinase (RTK), TrkA is NGF dependent and furthermore the association is time dependent following nerve growth factor (NGF) stimulation [9]. To study the impact of the CMT2B associated Rab7 mutants on TrkA interaction, we generated the GFP-tagged versions of Rab7 bearing L129F, K157N, N161T and V162M substitutions. Based on our previous studies, GFP-Rab7 fusion proteins preserve the functional properties of the untagged proteins [14]. As shown in Figure 1A, all of the CMT2B associated Rab7 mutants were observed to accumulate with a perinuclear distribution consistent with localization to endosomes. The level of expression of GFP-tagged Rab7 wt and mutant proteins in transfected PC12 cells was ∼3-fold higher than the endogenous Rab7 with transfection efficiencies of ∼35% (Figure S1A–S1B). In agreement with the published literature [9], endogenous Rab7 was found to co-immunoprecipitate with TrkA following NGF stimulation and the maximum interaction occurred 1 h after stimulation (Figure 1B). Therefore, we used 1 h NGF stimulation to test for an interaction between TrkA and GFP-tagged wild-type Rab7 as well as the CMT2B GFP-Rab7 mutants. GFP-tagged wild-type Rab7 and the Rab7 CMT2B mutants were all seen to co-immunoprecipitate with TrkA at similar levels (Figure 1C-1D), indicating that the mutant proteins were not impaired in their ability to bind to TrkA receptor.

### Rab7 CMT2B mutants expressing PC12 cells show differential TrkA phosphorylation following NGF stimulation

**Figure 2:**
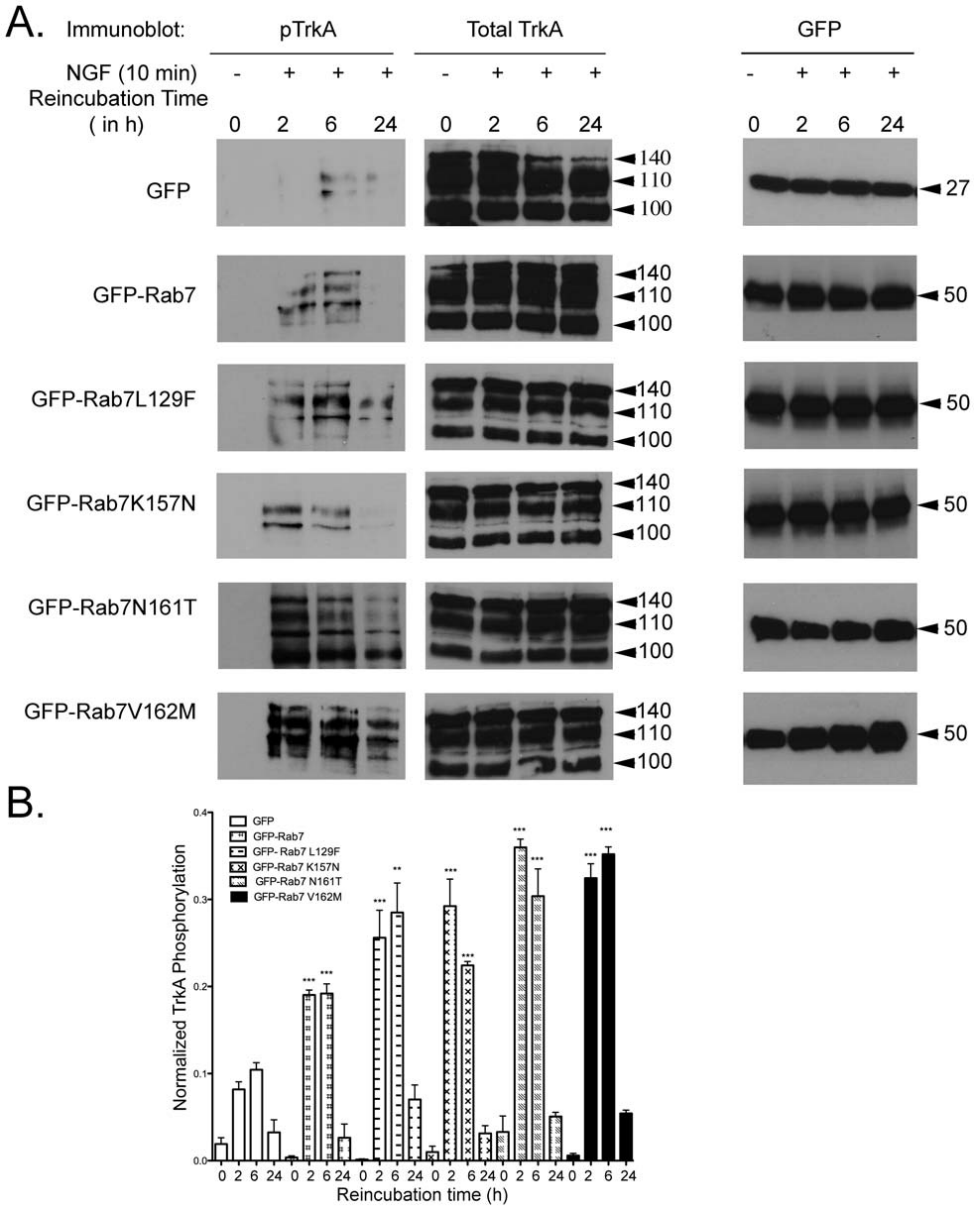
Cells Expressing Rab7 CMT2B Mutants Exhibit Higher Levels of Phospho-TrkA Following NGF Stimulation. (A) Transiently transfected PC12 cells expressing GFP, GFP-Rab7wt, GFP-Rab7L129F, GFP-Rab7K157N, GFP-Rab7N161T, GFP-Rab7 V162M were briefly stimulated with NGF (200 ng/ml) followed by surface stripping of ligand and reincubation in 0.1% BSA-DMEM for 2 h, 6 h and 24 h respectively. Subsequently, cells were lysed and equal amounts of protein were immunoblotted and probed for total and phosphorylated TrkA. Significantly, higher levels of phosphorylated TrkA were found at 2 h and 6 h. Equal amounts of protein from cell lysates were probed with antibodies against GFP. GFP-Rab7wt and GFP-Rab7 CMT2B mutant proteins are uniformly expressed in PC12 cells under these conditions as shown in the adjacent representative blot. (B) Films from four independent experiments were quantified by Image J analysis. Bar graphs show pTrkA in GFP-Rab7wt and CMT2B mutant expressing cells. In each case the amount of pTrkA was normalized to the amount of GFP protein expressed. The bar graph shows the fold-change in pTrkA as a function of pTrkA in the GFP only control at each respective time point. Error bars indicating mean±S.E.M., n = 4, **p<0.01, ***p<0.001. For detailed numerical values see Table 1.

Rab7 is known to control endosomal trafficking of TrkA as well as receptor signaling. To assess the effect of the Rab7 CMT mutants on NGF signaling, PC12 cells expressing the mutant proteins were briefly stimulated with NGF followed by removal of residual surface bound NGF (‘surface stripping of ligand’ as described in Materials and Methods) to maximize detection of signaling from activated, internalized TrkA receptors over the course of 2–24 h. Higher phospho-TrkA levels were consistently observed across multiple time points in cells expressing the GFP-Rab7 CMT mutants as compared to GFP-Rab7 wild-type or GFP only expressing control cells (Figure 2A). Expression levels of all GFP proteins were constant at all time points. Quantification of replicate experiments showed that Rab7L129F, Rab7K157N, Rab7N161T and Rab7V162M expressing cells had 2.1–3.9 fold higher levels of phospho-TrkA than the GFP only expressing control cells and 1.3–1.8 fold higher levels than the GFP-Rab7 wild-type expressing cells at the 2 h and 6 h time points (Figure 2B, numerical values are provided in Table 1). At the 24 h time points, the pTrkA signal was greatly diminished and the differences between the mutants and the controls were no longer statistically significant. There was no down regulation of total TrkA levels in the lysates, similar to previous reports where Rab7 dominant negative mutant was expressed [9]. Nevertheless, the higher TrkA phosphorylation is suggestive of differential interaction of the Rab7 mutants with signaling complexes that leads to an increase in activated TrkA receptors in endosomes.

**Table 1:** Fold-changes in phospho-TrkA and phospho- Erk1/2 due to Rab7 CMT2B mutants.

Expressed Protein	Phospho-TrkAFold change relative to GFP			Phospho-TrkAFold change relative to GFP-Rab7wt			Phospho-Erk1/2Fold change relative to GFP			Phospho-Erk1/2Fold change relative to GFP-Rab7wt		
	2 h	6 h	24 h	2 h	6 h	24 h	2 h	6 h	24 h	2 h	6 h	24 h
GFP	1.0	1.0	1.0				1.0	1.0	1.0			
GFP-Rab7 wt				1.0	1.0	1.0				1.0	1.0	1.0
GFP-Rab7L129F	3.1p<0.001	2.72p<0.01		1.34p<0.05	1.48p<0.05		2.58p<0.001	2.15p<0.01	2.67p<0.05	1.62p<0.01	1.75p<0.01	2.17p<0.05
GFP-Rab7K157N	3.57p<0.001	2.14p<0.001		1.53p<0.01	1.52p<0.05		2.49p<0.001	2.14p<0.001	2.84p<0.05	1.57p<0.001	1.74p<0.01	2.3p<0.05
GFP-Rab7N161T	4.39p<0.001	2.9p<0.001		1.88p<0.001	1.58p<0.01		2.98p<0.001	1.82p<0.01	2.54p<0.05	1.87p<0.01	1.48p<0.01	2.06p<0.05
GFP-Rab7V162M	3.96p<0.001	3.37p<0.001		1.70p<0.001	1.83p<0.001		3.14p<0.001	2.6p<0.001	2.65p<0.05	1.97p<0.001	2.12p<0.001	2.15p<0.05

Data from 4 independent experiments measuring TrkA phosphorylation and 3 independent experiments assessing Erk1/2 phosphorylation were evaluated using unpaired, one-tailed Student's t-test.

### Rab7 CMT2B mutants expressing PC12 cells show enhanced ERK signaling on NGF stimulation

**Figure 3:**
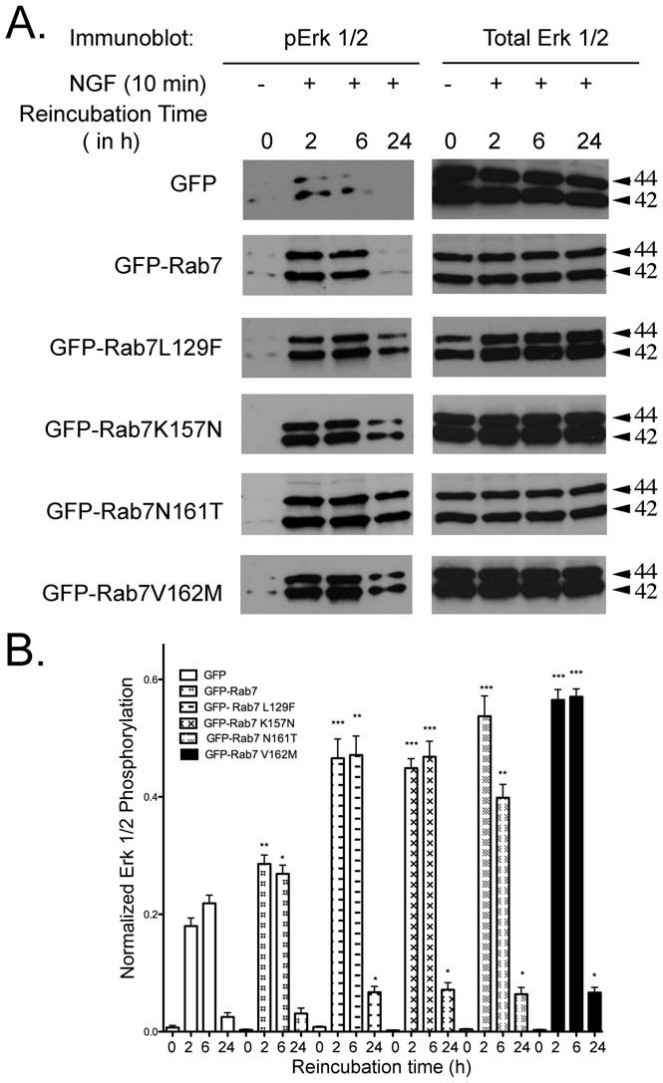
Cells Expressing Rab7 CMT2B Mutants Exhibit Enhanced Erk Signaling Following NGF Stimulation. (A) Transiently transfected PC12 cells expressing GFP, GFP-Rab7wt, GFP-Rab7L129F, GFP-Rab7K157N, GFP-Rab7N161T, GFP-Rab7 V162M were briefly stimulated with NGF (200 ng/ml) followed by surface stripping of ligand and reincubation in 0.1% BSA-DMEM for 2–24 h. Subsequently, cells were lysed and equal amounts of protein were immunoblotted and probed for total and phosphorylated Erk1/2. (B) Films from three independent experiments were quantified by Image J analysis. Bar graph shows levels of pErk1/2 in GFP-Rab7wt and CMT2B mutant expressing cells. In each case the amount of pErk1/2 was normalized to the amount of GFP protein expressed. The bar graph shows the fold-change in pErk1/2 as a function of pErk1/2 in the GFP only control at each respective time point. Error bars indicating mean±S.E.M., n = 3,*p<0.05; **p<0.01, ***p<0.001. For detailed numerical values see Table 1.

Upon NGF stimulation, PC12 cells stop proliferating and start differentiating [18]. NGF-mediated TrkA signaling in PC12 cells typically activates Erk1/2 and Akt downstream signaling. The Erk activation module is located on endosomes [19]–[21] and is instrumental in NGF stimulated differentiation signaling from TrkA containing endosomes [22]. To investigate the activation of these signaling mediators, PC12 cells transiently expressing Rab7 CMT2B mutants were briefly stimulated with NGF followed by surface stripping of ligand. Compared to control cells expressing GFP only or GFP-Rab7 wild-type protein, PC12 cells expressing the GFP-Rab7 CMT2B mutants consistently showed 1.5–2.2 fold higher levels of phosphorylated Erk1/2 at all time points examined (Figure 3A–3B, numerical values are provided in Table 1), providing evidence for enhanced downstream signaling.

### Rab7 CMT2B mutants expressing PC12 cells show no significant changes in Akt phosphorylation on NGF stimulation

**Figure 4:**
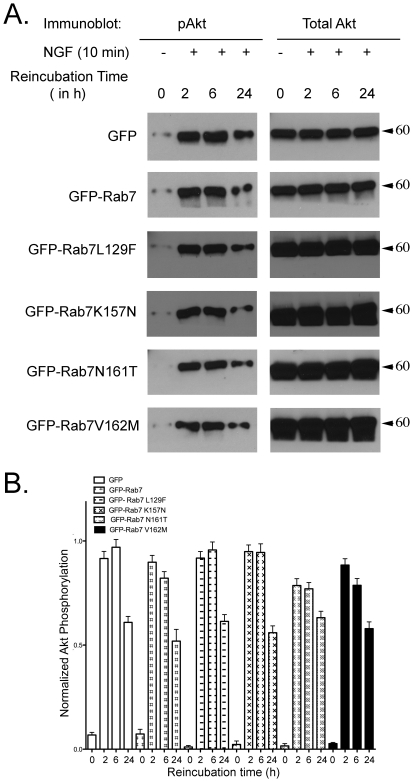
Cells Expressing Rab7 CMT2B Mutants Have no Effect on Akt Signaling. (A) Transiently transfected PC12 cells expressing GFP, GFP-Rab7wt, GFP-Rab7L129F, GFP-Rab7K157N, GFP-Rab7N161T, GFP-Rab7 V162M were briefly stimulated with NGF (200 ng/ml) followed by surface stripping of ligand and reincubation in 0.1% BSA-DMEM for 2–24 h. Subsequently, cells were lysed and equal amounts of protein were immunoblotted and probed for total and phosphorylated Akt. (B) Films from three independent experiments were quantified by Image J analysis. Bar graph shows levels of pAkt in GFP-Rab7wt and CMT2B mutant expressing cells. In each case the amount of pAkt was normalized to the amount of GFP protein expressed. The bar graph shows the fold-change in pAkt as a function of pAkt in the GFP only control at each respective time point. Error bars indicating mean±S.E.M. n = 3, p values were not significant, indicating no differences between samples.

The effect of NGF stimulation on the second major downstream signaling pathway regulated by phosphorylation of Akt was also analyzed in PC12 cells transiently expressing the Rab7 CMT2B mutants. There was no significant change in the Akt phosphorylation levels in the cells expressing the disease mutants when compared to cells expressing GFP alone or GFP-Rab7 (Figure 4A–4B). The activation of Akt was similar in all samples at early time points and diminished uniformly at 24 h. The findings are in agreement with a previous observation indicating that NGF-stimulated Akt signaling occurs at the cell surface and is not triggered on the endosomes [21]. The combined data suggest that alterations in TrkA downstream signaling are confined to endosomes and the Erk module, and are independent of Akt.

### Rab7 CMT2B mutants expressing PC12 cells show altered subcellular localization of phosphorylated Erk1/2 on NGF stimulation

**Figure 5:**
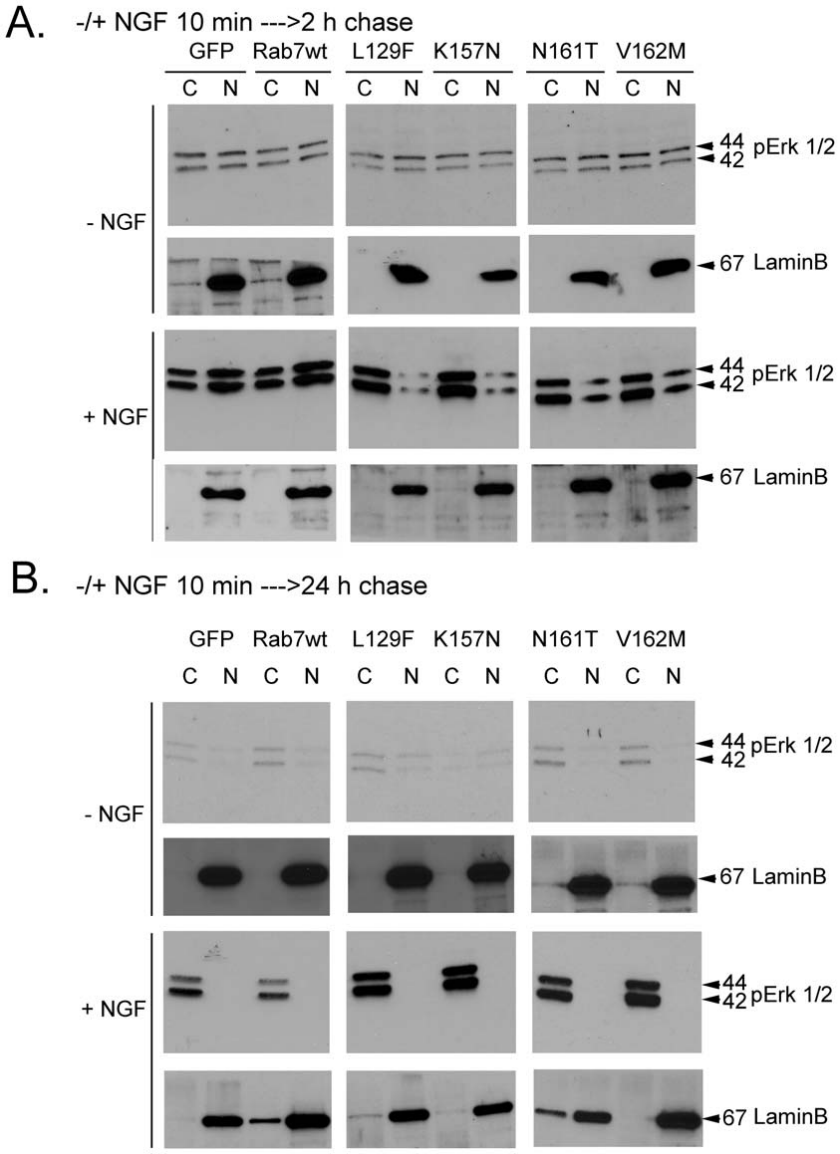
Cells Expressing Rab7 CMT2B Mutants Exhibit Altered Cytoplasmic and Nuclear Partitioning of Activated Erk1/2. Transiently transfected PC12 cells expressing GFP, GFP-Rab7wt, GFP-Rab7L129F, GFP-Rab7K157N, GFP-Rab7N161T, GFP-Rab7 V162M were briefly stimulated with NGF (200 ng/ml) followed by surface stripping of ligand and reincubation in 0.1% BSA-DMEM for (A) 2 h and (B) 24 h. Subsequently, cells were lysed and subjected to subcellular fractionation and immunoblotted with antibodies directed against phosphorylated Erk1/2. The purity of fractions was confirmed by probing for the nuclear marker lamin B.

To examine the subcellular distribution of activated Erk1/2 on NGF stimulation in PC12 cells we used subcellular fractionation to separate the cytosolic and nuclear fractions. Probing with lamin B, a nuclear envelope protein, tested the purity of the fractions. Following brief NGF stimulation and surface stripping of ligand, cells expressing the Rab7 CMT2B mutants were reincubated in starvation medium for 2 h and 24 h. At the shorter time point, the Rab7 CMT2B mutant expressing cells showed diminished levels of the activated Erk1/2 in the nucleus compared to GFP expressing control cells and consequently predominant cytoplasmic distribution of activated Erk1/2 (Figure 5A). However, after a 24 h post-stimulation chase period, activated Erk1/2 was no longer detected in the nuclear fraction in either the CMT Rab7 mutant or the wild-type Rab7 expressing cells (Figure 5B). Interestingly, the level of phosphorylated Erk1/2 remained higher in the cytosolic fraction of the disease mutants relative to cells expressing the wild-type Rab7, suggesting either enhanced phosphorylation or decreased dephosphorylation of the Erk1/2 in the cells expressing the disease mutants.

## Discussion

Charcot Marie Tooth Type 2B disease involves mutations in conserved amino acid residues of the late endocytic regulator Rab7. The four missense mutations of Rab7 implicated in the disease show similar biochemical properties that have been associated with the proteins being predominantly in GTP bound state [17], [23]. Here we demonstrate that the Rab7 CMT2B mutants enhance Erk signaling. This enhanced Erk signaling is likely to be dependent on the endosomal membrane residence time of the activated TrkA, which is in turn regulated by Rab7. Being predominantly in the GTP bound state the Rab7 CMT2B mutants would be expected to promote premature degradation of the TrkA containing signaling endosomes. However, it has been suggested that increased interaction of the CMT2B associated Rab7 mutants with various effector proteins notably Vps13C and ORP1L might result in an increase in the fraction of Rab7 CMT2B mutants that is active, but slow the transport of signaling endosomes and/or fusion with lysosomes by reducing temporal progression [16]. Consequently, the net effect of slowed trafficking of the activated TrkA caused by Rab7 CMT2B mutants present on endosomes is entirely consistent with the observed enhancement of phosphorylated Erk1/2 in PC12 cells.

Brief stimulation of PC12 cells expressing the disease causing Rab7 CMT2B mutants with NGF did not significantly affect Akt phosphorylation. There have been reports of the presence of activated Akt in various cell organelles such as nucleus, Golgi apparatus, cell surface and mitochondria, although not endosomes [24], [25]. Therefore, consistent with previous work showing Akt signaling predominates at the plasma membrane [9], [21], our data shows Akt signaling does not emanate from endosomes containing activated TrkA.

The strong persistence of phosphorylated Erk1/2 24 h after brief NGF stimulation was reported previously in PC12 cells expressing the GDP-bound Rab7T22N mutant [9]. The expression of the Rab7T22N mutant, which is stabilized in inactive state, did not however preclude nuclear localization of the activated Erk1/2. Furthermore, inhibition of Rab7 activity by overexpressing the Rab7T22N was shown to trigger neurite outgrowth in PC12 cells following brief NGF stimulation [9]. In contrast, we demonstrate that the Rab7 mutants associated with CMT2B led to accumulation of the phosphorylated Erk1/2 in the cytosol rather than the nucleus. This may explain the inhibitory effect of the Rab7 CMT2B mutants on neurite outgrowth as recently observed [10]. Sensory neurons have very long axons and endocytosis typically occurs at the distal axonal terminal. Signaling endosomes must therefore traverse a long way to the cell body via retrograde transport and consequently misregulation of the Rab7 cycle under diseased conditions (e.g. mutant versions of Rab7) would lead to altered trafficking and deficits in the translocation of activated Erk1/2. This could account for the more pronounced effects of these mutants in cells bearing longer neurites (>50 µm) [10]. The accumulation of activated Erk1/2 in the cytoplasm suggests that the CMT2B associated mutants retard kinase shuttling to the nucleus, which is required to upregulate the genes responsible for differentiation of sensory neurons. The data shed new light on the mechanism mediating the deficits in neurite outgrowth in Charcot-Marie-Tooth disease.

An intriguing question that remains is the role played by the MAP kinase phosphatases (MKPs) in regulating the level of phosphorylated Erk1/2. MKP3 expression is triggered by the translocation of the activated Erk to the nucleus, is maximal around 3 h after NGF stimulation and the upregulation can last as long as five days in PC12 cells [26]. MKP3 is cytoplasmic and acts specifically on Erk [27]. Because the disease associated Rab7 CMT2B mutants lead to a significantly higher levels of activated Erk1/2, the negative regulatory function that is normally met through the MKP activity is likely compromised in CMT2B disease. Our results suggest that the impaired neurite growth, which is an important disease phenotype, may be due in part to retardation of shuttling of the phosphorylated Erk1/2 to the nucleus, which is normally a prerequisite for upregulation of MKP3 expression and down regulation of Erk signaling [18].

In conclusion, our study throws light on the pathogenic mechanism leading to Charcot Marie Tooth Type2B disease by defining the enhanced Rab7-dependent activation of TrkA and downstream Erk signaling pathways.

## Materials and Methods

### Cells and reagents

PC12 and BHK-21 cells were from American Type Culture Collection (Manassas, VA). PC12 cells were grown in DMEM supplemented with 10% horse serum, 2.5% FBS, 2 mM glutamine, 50 U/ml penicillin and 50 µg/ml streptomycin and BHK-21 were grown in complete G-MEM (10% fetal calf serum, 2 mM glutamine, 50 U/ml penicillin, 50 µg/ml streptomycin, and 2.6 mg/ml tryptose phosphate broth). Cell culture reagents were purchased from Invitrogen/Gibco (Carlsbad, CA). Restriction and modification enzymes were from New England Biolabs (Ipswich, MA) and all chemicals used were from Sigma-Aldrich (St. Louis, MO). Recombinant NGF was from Invitrogen.

### Mutagenesis and plasmid construction

*Rab7a* used in these studies was *Canis lupus familiaris* (NM_001003316) [28]. GFP-tagged Rab7 CMT2B mutants (L129F, K157N, N161T and V162M) were constructed by site directed mutagenesis of wild-type *GFP-Rab7* in the pEGFP-C3 vector. The plasmids were used as templates for PCR-based mutagenesis. All amino acid substitutions were generated by a one-step reverse cyclic PCR method using the appropriate base changes in the synthetic oligonucleotides [29]. The primer sequences used for generating the mutants were as follows:

Rab7L129F (Fwd-5′-ATTGACTTCGAAAACAGACAAGTGGC-3′, Rev-5′-CTTGTTTCCCAACACAACGAAAGGGA-3′),

Rab7K157N (Fwd-5′AGTGCCAACGAGGCCATCAATGTGG-3′, Rev-5′-GGTCTCGAAGTAGGGAATGTTGTTT-3′),

Rab7N161T (Fwd -5′-GCCATCACCGTGGAGCAGGCGTTCCA-3′, Rev -5′-CTCCTTGGCACTGGTCTCGAAGTAGG-3′ and

Rab7V162M (Fwd -5′GCCATCAATATGGAGCAGGCGTTCCA-3′ and Rev -5′-CTCCTTGGCACTGGTCTCGAAGTAGG-3′). Mutations were confirmed by sequencing the entire Rab7 gene.

### Transient Transfection and Expression of Rab7 CMT2B Mutants

Cell lines were cultured as described above and passaged on consecutive days to maintain in logarithmic growth phase immediately prior to transfection. Transfections of PC12 cells were performed using Lipofectamine 2000 (Invitrogen) according to manufacturer's instructions. Rab7 expression was maximal 16–24 h post-transfection and experiments were conducted during this time frame. Transfection efficiency of ∼35% was consistently observed.

### NGF stimulation protocol

NGF (200 ng/ml) stimulation was performed as described previously [9].

### Antibodies

Rabbit polyclonal antibody directed against Rab7 was used for immunoblotting, and immunoprecipitation assays as described [7], [30]–[32]. The following commercial antibodies were used: mouse mAb directed against ERK1/2, mouse mAb directed against phospho ERK1/2, rabbit pAb directed against phospho TrkA, rabbit pAb directed against Akt, mouse mAb directed against phospho Akt, β-actin rabbit mAb HRP conjugate, all from Cell Signaling Technologies (Beverly, MA); rabbit pAb directed against GFP from Invitrogen (Carlsbad, CA), mouse mAb directed against Rab7 from Sigma, goat pAb directed against lamin B from Santa Cruz Biotechnology, Inc. (Santa Cruz, CA), rabbit pAb directed against TrkA from Millipore Inc. (Billerica, MA)

### Immunoprecipitations

Sixteen hours (h) post-transfection, PC12 cells were washed in PBS and transferred to DMEM/BSA, starved for 5 h and stimulated with NGF for 10 min, followed by surface stripping of ligand with ice cold stripping buffer (0.2 M acetic acid and 0.5 M NaCl) [33]. Cells were subsequently washed thrice in ice cold PBS and reincubated in serum free medium for the various times (2–24 h). Cells were washed in PBS and lysed for 15 min on ice in lysis buffer (0.5% NP40, 25 mM Tris-HCl, 100 mM NaCl, 50 mM NaF, 1 mM Na_3_VO_4_, pH 7.5 supplemented with protease inhibitor mixture from Calbiochem/EMD Chemicals, Gibbstown, NJ). Lysates were precleared by centrifugation at 13,000 rpm in an Eppendorf microcentrifuge for 10 min and pretreatment with protein A Sepharose (Amersham Biosciences/GE Healthcare, Piscataway, NJ); 500 µg cell lysate/40 µl protein A Sepharose at 4°C for 3 h with mild rotation. The supernatant (precleared lysate) was transferred in a clean microfuge tube and incubated with 3 µg rabbit pAb directed against GFP (Invitrogen) for 2 h at 4°C. Antibody complexes were recovered by incubation with protein A Sepharose (40 µl) at 4°C for 3 h. Immunoprecipitates were washed in 0.1% NP-40, 25 mM Tris-HCl, pH 7.5, 150 mM NaCl, 1 mM Na_3_VO_4_ supplemented with protease inhibitor and a final wash of ice cold, 10 mM Tris-HCl, and pH 7.5. The beads were boiled in Laemmli buffer and the proteins resolved by SDS-PAGE (10% gel).

### Subcellular Fractionation

Nuclear and cytosolic fractions were prepared as previously described with minor modifications [34]. PC12 cells were plated on 60 mm dishes and transfected to express the respective GFP-tagged Rab7 wild-type or CMT disease mutants. To separate the nuclear and cytosolic pools of Erk1/2, the cells were serum starved for 5 h and stimulated with NGF for 10 min followed by surface stripping of the ligand. The cells were then incubated in starvation medium (DMEM containing 0.1% BSA) for 2 h, 6 h or 24 h at 37°C. The cells were washed thrice in ice cold PBS and scraped in 750 µl of lysis buffer (10 mM Tris-HCl, pH 7.4, 10 mM NaCl, 3 mM MgCl_2_ 0.3% (v/v) NP-40, 1 mM PMSF, 1 mM Na_3_VO_4_, 10 mM NaF, 30 mM sodium β-glycerophosphate, 10 µg/ml chymostatin, 10 µg/ml leupeptin, 10 µg/ml antipain and 10 µg/ml pepstatin). Cells were incubated on ice for 5 min followed by centrifugation at 500×g for 5 min to pellet the nuclei. The cytosolic fraction separated in the supernatant. Nuclei pellets were washed in the same lysis buffer without NP40. The cytosolic and nuclear fractions were boiled in 2× Laemmli sample buffer and resolved on a 10% SDS polyacrylamide gel and probed with a phospho Erk1/2 antibody. The purity of the nuclear fraction was checked with an antibody directed against nuclear lamin B.

### Confocal Immunofluorescence microscopy

BHK21 cells were grown and processed for immunofluorescence staining using standard procedures. Briefly, transfected cells overexpressing Rab7 GFP and the Rab7 CMT2B mutants GFP were pre-permeabilized with 0.05% (v/v) saponin in PIPES buffer (80 mM PIPES-KOH, pH 6.8, 5 mM EGTA, 1 mM MgCl_2_) for 2–3 min, to allow optimal visualization of membrane structures and minimize cytosolic protein staining, and then fixed with 3% (w/v) paraformaldehyde in PBS. The coverslips were washed four times with PBS/saponin and mounted on glass slides in Mowiol 4–88 [32]. Fixed cells were imaged on a Zeiss LSM510 confocal microscope equipped with a 63× oil immersion lens.

### Statistical Analyses

The experiments were performed at least three times and statistical data are shown as mean±S.E.M. Differences were analyzed by unpaired, one-tailed Student's *t* test using GraphPad Prism software.

## Supporting Information

Figure S1 **GFP‐Rab7wt and Rab7 CMT2B Mutant Proteins are Uniformly Expressed in PC12 Cells 3‐Fold above Endogenous Levels.** (A) Transiently transfected PC12 cells expressing GFP, GFP‐Rab7wt, GFP‐Rab7L129F, GFP‐Rab7K157N, GFP‐Rab7N161T and GFP‐Rab7 V162M were lysed 16 h post‐transfection and immunoblotted for Rab7 to probe for GFP tagged proteins as well as the endogenous Rab7 levels. (B) Films from three independent experiments were quantified using Image J analysis. Values of overexpressed (OE) GFP‐Rab7wt and GFP‐Rab7 CMT2B mutant protein expression were compared to endogenous (Endo.) Rab7levels. Error bars indicating mean ±S.E.M. n=3, *p<0.05; **p<0.01.
(TIF)
