# Supplementary figures and images for: Rab7 Mutants Associated with Charcot-Marie-Tooth Disease Exhibit Enhanced NGF-Stimulated Signaling
Source: PLoS One. 2010 Dec 9;5(12):e15351. doi: 10.1371/journal.pone.0015351 (PMC3000344; doi:10.1371/journal.pone.0015351)

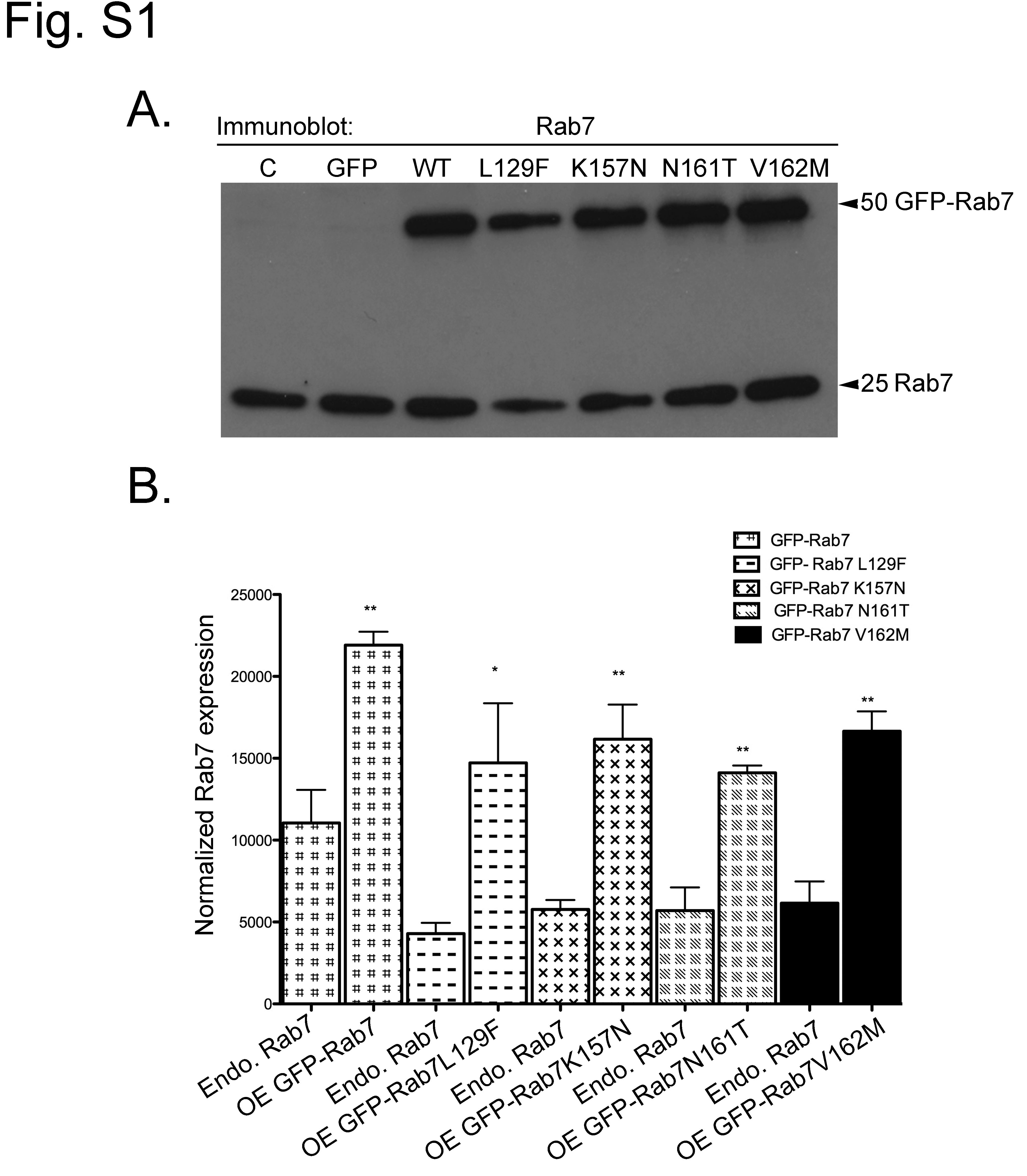

Supplement: Figure S1 — GFP‐Rab7wt and Rab7 CMT2B Mutant Proteins are Uniformly Expressed in PC12 Cells 3‐Fold above Endogenous Levels. (A) Transiently transfected PC12 cells expressing GFP, GFP‐Rab7wt, GFP‐Rab7L129F, GFP‐Rab7K157N, GFP‐Rab7N161T and GFP‐Rab7 V162M were lysed 16 h post‐transfection and immunoblotted for Rab7 to probe for GFP tagged proteins as well as the endogenous Rab7 levels. (B) Films from three independent experiments were quantified using Image J analysis. Values of overexpressed (OE) GFP‐Rab7wt and GFP‐Rab7 CMT2B mutant protein expression were compared to endogenous (Endo.) Rab7levels. Error bars indicating mean ±S.E.M. n=3, *p<0.05; **p<0.01. (TIF) [file pone.0015351.s001.tif]
